# Visual perception of shape altered by inferred causal history

**DOI:** 10.1038/srep36245

**Published:** 2016-11-08

**Authors:** Patrick Spröte, Filipp Schmidt, Roland W. Fleming

**Affiliations:** 1 Justus-Liebig-University Giessen, Department of General Psychology, Otto-Behaghel-Strasse 10F, D-35394 Giessen, Germany

## Abstract

One of the main functions of vision is to represent object shape. Most theories of shape perception focus exclusively on geometrical computations (e.g., curvatures, symmetries, axis structure). Here, however, we find that shape representations are also profoundly influenced by an object’s causal origins: the processes in its past that formed it. Observers placed dots on objects to report their perceived symmetry axes. When objects appeared ‘complete’—created entirely by a single generative process—responses closely approximated the object’s geometrical axes. However, when objects appeared ‘bitten’—as if parts had been removed by a distinct causal process—the responses deviated significantly from the geometrical axes, as if the bitten regions were suppressed from the computation of symmetry. This suppression of bitten regions was also found when observers were not asked about symmetry axes but about the perceived front and back of objects. The findings suggest that visual shape representations are more sophisticated than previously appreciated. Objects are not only parsed according to what features they have, but also to how or why they have those features.

One of the most important functions of vision is to infer and represent the shape of objects. Shape perception is crucial for many tasks including object recognition^1^,^2^,^3^,^4^, guiding reaching and handling actions^5^,^6^,^7^,^8^, and making other high-level inferences about object properties, such as whether an object is physically stable or likely to topple over^9^,^10^,^11^. However, estimating and representing object shape from the retinal image is far from trivial^1^,^3^,^4^,^12^,^13^,^14^,^15^,^16^.

To make matters worse, inferring the geometrical properties of local surface patches—such as their depth, orientation or curvature—is only the start of the shape-inference process. It is clear that there is much more to shape perception than making local judgments about surface structure. In order to make higher-level inferences about objects (e.g., identifying symmetry axes, identifying front and back ends, predicting likely physical weight, or parsing the object into functional parts, like the cup and handle of a mug), the brain must somehow pool and organize information from distant locations across the object into more global quantities—a process known as perceptual organization^17^,^18^. For example, to work out if an object is symmetrical, it is necessary to compare how corresponding object features co-vary relative to a symmetry axis, while factoring out other potential match locations^19^,^20^.

Almost all work to date has focused on how perceptual organization depends on the geometrical properties of shapes, for example, when explaining how shapes are decomposed into parts^21^,^22^,^23^ or how missing pieces of occluded or fragmented shapes are interpolated^24^,^25^,^26^,^27^,^28^,^29^,^30^,^31^,^32^,^33^,^34^. Here, however, we are interested in how shapes are perceived and represented depending on higher-level inferences about the causal origin of objects and their features. Consider, for example, the cookie and croissant in Fig. 1: despite the geometrical similarity of the 2D shapes, we readily assign different physical causes to their concavities. The cookie is bitten while the croissant is bent. Somehow, by combining perceptual shape computations with experiences from our past, we are able to infer the causal origins of the two different kinds of concavity.

Leyton^35^,^36^ was one of the first to suggest that this inference of causal origin plays a role in the visual perception of shape (also see ref. 37). He provided a framework in which simple shape features are interpreted in terms of causal attribution. Specifically, forces that shape an object are thought to operate along local symmetry axes within the shape contour. These axes are defined by and extend into the curvature extrema along the contour (e.g., into the tips of the cookie or croissant in Fig. 1). Consequently, the presence of an extremum indicates that a force has acted on a shape and its direction indicates the direction of that force. Although Leyton’s framework is potentially powerful in capturing the visual representation of causal origin, it cannot explain differences in causal attribution for objects that are geometrically very similar (Fig. 1).

Figure 2 further illustrates the deep connection between shape representation and causal inference. The shape in the top row appears to be a gently curved object consisting of a single trunk. However, a subtle change in the object’s shape—roughening the concavity on the right side—completely changes the interpretation of the causal history of the object (bottom row). Instead of consisting of a single limb, it looks more like it was originally a three-pronged object, one of whose limbs has been removed. Again, tiny differences in retinal terms can yield substantially different subjective impressions of their overall organization and structure, depending on the inferred causal origin of the features.

Together, this suggests that the brain can to some extent infer the causal origin of specific shape features of objects, and take this causal origin into account when parsing and representing shape^35^,^36^,^38^. These inferences may not be purely perceptual in nature. Indeed, previous studies demonstrated effects of semantic knowledge on other perceptual phenomena, such as amodal completion of partially occluded objects^39^ or object segmentation^40^. Consequently, when the causal origin of features is ambiguous, observers may be able to entertain multiple distinct percepts at will. Here, however, we do not seek to pin down the relative contributions of bottom-up information and top-down knowledge in the process of integrating causal origin into the representation of shape (although that is of course an important topic). We reason that before such questions can be answered, we must first investigate whether causal inferences affect judgments of shape properties per se. If observers derive different perceptual organizations from geometrically very similar shapes depending on the apparent origin of their features, this would suggest that causal inference is indeed an important aspect of visual object perception.

To test this idea, we have developed two intuitive tasks in which observers place dots either along the perceived principal axes of shapes (Experiments 1 and 2) or at the front, back and center of shapes (Experiment 3) in order to report what they believe to be the natural organization of the shape. We find that both observers’ symmetry axis judgments and front-back judgments are substantially influenced by higher-level information about object shape, including familiarity and inferred causal processes. Participants appear to parse unfamiliar objects into portions with distinct causal origins and suppress features that are caused by bites when computing either object symmetry or front and back. Indeed, we find that whether two objects appear to have similar symmetry axes or similar fronts and backs depends more on the inferred causality of the objects than on their geometrical similarity in the image.

## Experiment 1

### Results and Discussion

To test whether causal inferences can influence the perceptual organization of shape, we created a set of 2D objects with different causal histories (Fig. 3), and asked observers to indicate where they perceived the central axis of the objects, by freely distributing ten dots along the axis’ length.

If the perception of shape in general—and global axis in particular—is unaffected by generative interpretations of the shapes’ parts, we would expect observers’ responses to rely solely on geometrical factors (e.g., the curvature or extent of a concavity), irrespective of the apparent causal origins of the features (e.g., whether a concavity appears to be due to a bite or a bend). Such geometry-based responses may be partly approximated by the medial axis of the shape, or other related shape skeleton representations, which derive an object’s local symmetry axes from its boundary^13^,^15^,^41^,^42^. In contrast, if the interpretation of generative processes does influence judgments, different shape transformations should lead to different perceptual organizations, which in turn should show up as different responses to certain object features. For example, when a concavity is perceived as being due to a distinct causal process (e.g., bitten out of the shape) so that it does not appear to ‘belong’ to the rest of the object, we may expect the participants to exclude (or downplay) the concavity from judgments of the object’s central axis.

Figure 3 shows pooled responses of all participants to the ten shapes (black dots). As can be seen, the responses are generally quite consistent across participants, tending to cluster into one or two clear axes for each shape. In most cases these were straight lines, but when the object as a whole appeared curved (shapes 4 and 5), participants also readily traced curved axes along the centre of the shape. More importantly, the data also indicate that inferences about the causal origins of shape features can affect perceptual organization judgments. The top row (shapes 1–5) shows objects that appear complete, while the bottom row (shapes 6–10) shows objects that appear bitten. Where a concavity in the outline appears to be caused by a bite (e.g., the apple), observers can suppress or exclude the concavity from their judgments about the locus of the object’s central axis. The observers readily reported the axis that would correspond to the shape of the complete version of the object, as if they explicitly compensated for the missing portion of the object. This suggests that the visual estimation of symmetry axes is a ‘robust’ computation, which either includes or excludes certain features of the object depending on whether they are interpreted as being significant, defining features of the object, or incidental (i.e., caused by some extrinsic process).

To characterize this observation more formally, we fitted the data using non-linear PCA (red lines; Matlab Toolbox developed by ref. 43) and compared these fits to a prediction derived from the contours of the shapes (shape skeleton; green lines). As can be seen, the red response fits deviate substantially from the green predictions based on local geometry. Indeed, for the bitten objects, participants approach or even cross the contour portion created by the biting process. This suggests that measures of central axis based purely on local geometry cannot explain observers’ performance in the task.

Rather, we propose that when observers identify a shape feature as being caused by a bite, they mentally complete the missing portion of the shape, much like the amodal completion of partially occluded objects^29^,^44^,^45^,^46^. Thus, when asked to report the central axis of a bitten object, their results are close to the axis for the completed form (blue lines in Fig. 3), thereby robustly excluding the bite from their axis judgments. Taken together these results suggest that inferences about generative shape processes (here a bite transformation) can alter processes of perceptual organization.

These results provide some initial evidence for an effect of causal interpretations on perceptual organization. However, in Experiment 1 the comparison between bitten and complete objects co-varied with the identity of the shape (e.g., kidney vs. bitten rectangle), so that the objects with different interpretations were geometrically very different from one another. As a result, we cannot measure the magnitude of the effect of causal interpretations, which requires comparing bitten and complete versions of the same object. Moreover, most of the objects were familiar and mirror symmetric, which presumably facilitated the estimation of symmetry axes for the bitten objects. Thus, to test the generality of our findings, we conducted a second experiment.

## Experiment 2

We sought to test whether observers compensate for extrinsic concavities due to excision, but do not compensate for concavities in general. We created a set of irregularly shaped asymmetric objects exhibiting intrinsic concavities whose local symmetry axes did not coincide with global symmetry axes and that were unfamiliar to observers.

To do this, we created several versions of each object (see Fig. 4A for some examples), so that the pattern of responses could be directly compared across conditions: (i) complete shapes, (ii) bitten versions which were derived from the complete objects by excising a portion of the shape, thereby introducing a concavity with a jagged outline, and (iii) smoothed versions of the bitten objects which contained a similar concavity, but where the contour of the concavity had been smoothed to make it appear to be an intrinsic feature of the object and not due to a bite.

As a result, in retinal image terms, the bitten and smoothed versions of the same objects were more similar to one another than to the complete object. However, in terms of the perceived organization of the parts and features of the shape, the bitten version of the objects tended to appear more similar to the complete objects than to their smoothed counterparts. We documented these effects experimentally using the same dot placement task.

### Results and Discussion

Rating results (Fig. 4B) verify that the causal properties of the shapes were perceived as intended: both the complete and smoothed shapes were not seen as bitten, whereas the bitten shapes were. Shapes that were created by the biting process received higher values on the bitten dimension (mean = 74, SEM = 1.04) than complete (mean = 22, SEM = 0.89) or smoothed (mean = 25, SEM = 0.99) shapes, which in turn received higher ratings on the smooth dimension (mean = 53 and 51, SEM = 2.68 and 2.19, respectively). None of these stimuli appeared strongly rough, suggesting that observers interpret the jagged outline as being due to a bite.

In the dot placement task, in contrast to the responses in the control of Experiment 1 (Fig. S1), we found that observers did not spontaneously compensate for the bitten regions without further instructions. When confronted with an asymmetric bitten shape, the majority of observers asked whether they should indicate the axis spanning the removed region of the shape or whether they should report an axis that lies within the shape contours. Evidently, the presence of a feature with a distinct causal origin causes participants to question how the different regions should be combined when asked to perform a global perceptual organization task. Put differently, because observers are generally unfamiliar with explicitly reporting global shape axes, when confronted with unfamiliar, asymmetric shapes they were uncertain about the type of response to give. This uncertainty can be reduced either automatically by symmetry and top-down knowledge (as in the control of Experiment 1), or by explicit verbal instructions. Here, we told them that if they saw a given region as being bitten, they should place the axis where it would lie if they saw the original, unbitten form of the shape. Importantly, we did not inform them *whether* any given shape should be seen as bitten or not, nor where on the shapes the bites were located. We find that when instructed this way, observers readily identify and mentally undo the bite process. Overall responses to complete shapes (Fig. 4A, left column) follow smooth, approximately central axes (represented by the first component of non-linear PCA of observers’ responses). By contrast, responses to bitten shapes approached or even crossed the excised regions, indicating that observers could distinguish and compensate for distinct causal contributions to the shape. Importantly, the reported axes only approach or cross into those concavities that appear to be extrinsic to the shape (i.e., bites) but not those that appear to be intrinsic.

To quantify the effects of causal inference on the participants’ responses, we measured the similarity between the fitted and physical axes. Individual bitten and smoothed versions were derived from the same complete shape, therefore differences in the form and location of their principal axes could only result from differences in the transformation.

Building on our findings in Experiment 1 that observers compensate for excised portions of a shape, we should expect curves derived from bitten shapes to be more similar to the curves derived from complete shapes, than to those derived from the smoothed objects. The Fréchet distances^47^ between points along those curves confirm this (see also Fig. S2 for superposition of derived axes). Responses to bitten shapes were more similar to complete shapes (mean distance = 26.92, SEM = 6.25) than to the smoothed versions (mean = 41.27, SEM = 14.08) for all shapes except 1 and 6 (Fig. 4C: orange dots to the right of the red dashed line). In all cases, the reported axes for the bitten shapes were nevertheless more similar to those of the complete objects than the physical outlines were (Fig. 4C: orange dots to the left of the blue dots).

Taken together, the results of Experiment 2 indicate that when a concavity was perceived to be due to excision (bite transformation), observers could locate and compensate for the missing portion, leading to changes in the perceived organization of the shape.

However, with this task, observers did not compensate spontaneously for bites in their responses but only after explicit verbal instructions. Moreover, the responses in the dot placement task might have been influenced by the preceding rating task. Specifically, the rating might have primed them to interpret shapes as bitten and to respond accordingly in the dot placement task. To address these two concerns, we conducted a third experiment.

## Experiment 3

We used a variant of the dot placement task (front-back task) with a new set of stimuli to replicate the principal results of Experiments 2 without providing explicit verbal instructions. Instead of placing ten dots along the perceived symmetry axis, the observers were asked to place dots at the ‘front’, ‘back’, and ‘center’ of each shape. Stimulus ratings were obtained from a different group of observers to rule out possible interferences between the two tasks.

### Results and Discussion

Results are shown in Fig. 5 and Figs S3–5. The subjective ratings confirmed that observers interpreted the transformations as expected (Fig. 5B). Bitten shapes were rated highest on the bitten dimension (mean = 75.60, SEM = 3.67) and complete and smoothed shapes were rated highest on the neither (smooth) dimension (mean = 94.08, SEM = 0.84 and mean = 83.31, SEM = 2.46, respectively).

As a measure of response similarity in the front-back task, we plotted histograms of response frequency around the perimeter of the shape (Fig. 5A, second column). Correlations between these histograms are considerably higher for complete and bitten versions (r = 0.89) than for complete and smoothed versions (r = 0.38, z = 2.45, p = 0.014; 48) or for bitten and smoothed versions (r = 0.39, z = 2.43, p = 0.015). At the same time, the similarities in observer responses are in stark contrast to the physical similarity of the shapes; here, pixel-wise correlations are considerably lower for complete and bitten versions (r < 0.01) and for complete and smoothed versions (r < 0.01) than for bitten and smoothed versions (r = 0.93, both z = 3.51, p < 0.001). Thus, physically small changes to a shape, can quite substantially alter the perceptual organization of the shape as a whole when they induce the impression of a different causal origin. This replicates the findings of Experiment 2 in a related task without explicit verbal instructions and without any possible interference between the rating task and the dot placement task.

In the center task (Fig. 5A, third column), correlations between response histograms were not significantly different for complete and bitten versions (r = 0.45) and for complete and smoothed versions (r = 0.32, z = 0.63, p = 0.527) or for bitten and smoothed versions (r = 0.75, z = −1.10, p = 0.272). This results from the only marginal difference between the center of complete and smoothed versions (in shapes 2 and 3) or from a reluctance of many observers to place dots in the empty space of the bite (in shapes 1 and 4; see Fig. 5A and Figs S3–5).

The main results are summarized in Fig. 5C, which is based on the similarity between response histograms and pixel-wise similarity between shapes. Responses to bitten shapes in the front-back task were more similar to complete shapes than to the smoothed versions for all shapes (Fig. 5C: large orange dots to the right of the red dashed line). This was not the case for responses in the center task, although in every case they were shifted more towards the complete shapes than would be expected from the physical similarity of bitten and smoothed shapes (Fig. 5C: small orange dots to the left of the blue dots).

Our findings suggest that the visual estimation of front and back, like the visual estimation of symmetry axes, is a robust computation which includes or excludes object features depending on whether they are interpreted as being significant (intrinsic) or incidental (extrinsic). Importantly, in the front-back task this computation was carried out spontaneously without any explicit verbal instruction of the observers and without a preceding rating task, which might have prompted observers to think in terms of bitten objects.

**Figure 2:**
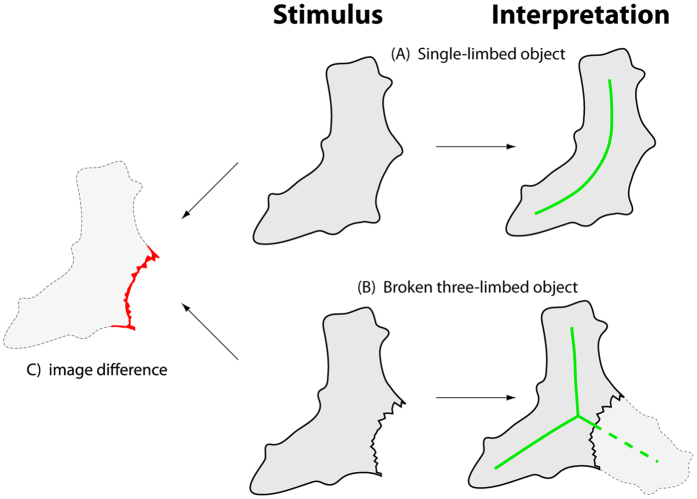
Example of shape representation from causal inference. Two versions of a shape that are similar in retinal terms but where different causal inferences result in different impressions of their overall structure—a complete single-limbed, slightly curved object (top row) and a broken, three-limbed, star like shape (bottom row). Green lines illustrate apparent axis structure.

Taken together, our results suggest that high-level inferences about the causal origin of shape features can influence the way the visual system pools and interprets those features into global shape representations. Sometimes small changes in the geometrical properties of an object can radically alter the interpretation of an object’s causal history. Changing this interpretation (Experiments 2 and 3) can have substantial consequences for perceptual organization—such as the object’s global axis (Experiment 2), the number of its parts (see Fig. 2), or perceived front and back (Experiment 3). This in turn may affect other inferences about the object, such as its material properties, class membership, or likely behavior, suggesting that generative models of shape play an important role in the visual and cognitive representation of shape.

### General Discussion

We have tested whether the human mind can infer the causal origins of specific shape features (e.g., concavities) and take those origins into account when parsing and representing shape. Our results suggest that the visual-cognitive system decomposes both familiar and unfamiliar objects into components with distinct causal origins (e.g., bites versus intrinsic concavities). These higher-order causal interpretations in turn influence the way in which shape symmetry, and the ‘front’ and ‘back’ ends of objects are computed. In particular we found that when reporting an object’s symmetry axis or its front and back, observers are able to suppress information about concavities that are caused by excision (i.e., bites). In contrast, no suppression occurred when the concavities were interpreted as intrinsic to the shape. Note that the relative importance of bottom-up and top-down factors in this process still has to be evaluated.

Intuitively, the presence of relatable contour parts^49^,^50^, and a concavity flanked by tangent or curvature discontinuities^51^, seem necessary for a bite interpretation. These are conditions similar to those that are known to induce amodal completion when a portion of the object is missing from the image due to occlusion behind another object. However, in contrast to amodal completion, where the border of the concavity is perceived as belonging to the occluder, border ownership does not reverse within concavities due to excision^52^,^53^. Moreover, in order to know whether completion should occur or not, the visual system must interpret the causal origin of the contour within the concavity. Only those concavities that are due to excision are suppressed from symmetry computations.

A comparable finding of high-level inferences from shape features was reported by Sigurdardottir, Michalak, and Sheinberg^54^. The authors asked participants to judge the orientation of a large set of unfamiliar random shapes and found that most shapes had an inherent directionality, which was reported by the majority of participants. In a series of experiments, Sigurdardottir *et al*.^54^ demonstrate how this directionality guides spatial attention, increases accuracy of (direction-congruent) motion detection, and increases tracking performance when participants followed (direction-congruently) moving shapes with their eyes.

These results demonstrate that shape perception involves more than simply detecting local features (e.g., orientations, depths or surface curvatures) and then pooling them in a purely geometrical way into complete objects. Such a view disregards the highly flexible nature of perceptual organization, the potential involvement of cognition in shape representation, and the high-level computational challenges involved in parsing and representing shape, such as the attribution of causal significance to features.

Consequently, we suggest that in addition to purely geometrical processing, the visual system also seeks to infer the underlying generative processes that structure and organize the observed shape: the causes and processes that have endowed the shape with its specific features^35^,^36^,^37^,^38^. We have focused here on one specific example, related to the perception that objects are intact vs. incomplete. However, we suggest that our findings hint at a much broader set of processes of shape understanding, through which the visual system infers primitive generative models of shape, to account for objects’ salient features. When we look at an object, we not only work out what shape it has, but to some extent, how or why it has that shape by parsing and interpreting the geometrical structure of the object to identify its most important defining features and how they are related to one another. Inferring such generative models of objects from their shape would have many potential uses. For example, understanding the origins of shape features would facilitate inferences about other physical and functional properties of objects, such as their material properties (e.g., ductility, viscosity, fragility; see ref. 55), or how other members of the same objects class are likely to look.

Others have demonstrated that top-down information such as expectations^56^,^57^,^58^ and familiarity^58^,^59^,^60^,^61^,^62^ also play an important role in shape perception. Here, we extend this view by showing that inferences about the origin of shape features (e.g., transformations) can also alter perceptual organization and shape representation. Although others have emphasized the role of generative models in visual shape perception^3^,^4^,^23^,^35^,^41^,^63^, none of these models distinguishes between geometrically similar parts with different causal origins (e.g., whether a concavity is intrinsic or extrinsic). Moreover, there remains little in the way of experimental work on the visual inference—and use—of generative models of shape for material perception^64^, category learning^65^,^66^,^67^ and other complex tasks that lie at the intersection between vision and cognition. To what extent such tasks can be solved by simple feedforward pattern recognition architectures or whether they require more sophisticated internal models, including inferences about hidden processes, is a major open question for future research.

## Methods

### Experiment 1

#### Observers

13 observers participated in Experiment 1. Here and in the following experiments, the number of observers was chosen based on a previous study using a dot placement task^68^. Observers were recruited through the university’s internal mailing list. All participants reported having normal or corrected to normal vision and were naïve to the purpose of the task. Participation was compensated with 8€/hour. Experiments were conducted in accordance with the Declaration of Helsinki and experimental protocols were approved by the ethics board at Justus-Liebig-University Giessen LEK FB06 (SFB-TPC1, 09/11/13). All participants gave written informed consent prior to taking part.

#### Stimuli

**Figure 3:**
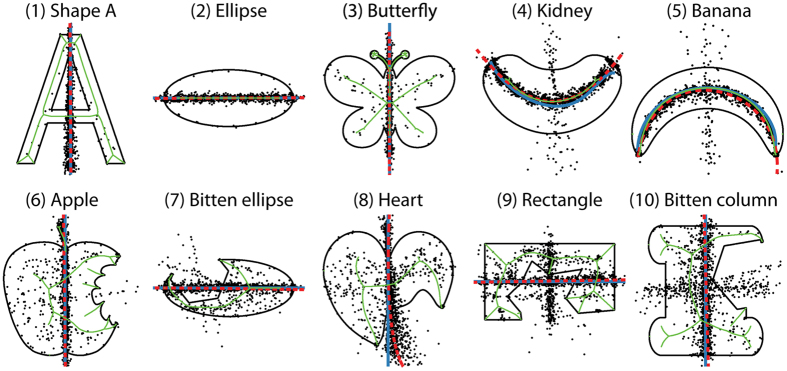
Stimuli and results of Experiment 1. Black dots (N = 1560 per shape) represent observers’ individual responses. Red dashed lines represent fits to the data points (perceived symmetry axes) using non-linear PCA. The complete shapes’ true symmetry axes (only complete shapes) and the axes of completed versions of the bitten shapes are colored in blue and were mathematically determined or in case of the kidney and banana shape derived using non-linear PCA. Green contours represent shapes’ skeletons computed using the Puffball algorithm^71^. Note that a small portion of the responses crossed concavities that were intrinsic features of the objects (e.g., in the kidney or the banana), reflecting the fact that objects can have more than one symmetry axis.

Stimuli were ten symmetric 2D shapes (see contours in Fig. 3). Five of the ten shapes had a part removed by subtracting another arbitrary shape (bite transformation). The length of the longest (vertical or horizontal) axis of all base shapes was 7.90° of visual angle (1 cm ~0.57° of visual angle). All stimuli are available for download at http://dx.doi.org/10.5281/zenodo.159923.

#### Procedure

Observers were seated about 1 m away from a Dell U2412M monitor at a resolution of 1920 × 1200 pixels. Stimuli were presented black on a white background controlled by Matlab using the Psychophysics Toolbox extension^69^. On each trial one of ten shapes was presented at a random orientation in the middle of the screen. Observers then indicated the symmetry of that same shape by placing ten dots along the perceived symmetry axis. Errors could be corrected by removing the dots in question and placing them at the desired location. Placing the last dot marked the end of a trial and initiated the judgment for the next shape. Instructions were provided in a written form and observers had to repeat them in their own words so that potential misunderstandings concerning the task could be clarified at an early stage. We used two different sets of instructions: either explicit instructions to place the dots along the axis of the complete object when they saw the shape as missing a portion (results reported in the following), or, in a different task, with no instructions referring to the interpretation of the whole or parts of the object (control with n = 6 observers; Fig. S1). The first instructions were chosen to keep consistent with Experiment 2 (see 3.1 Results and Discussion); the second instructions were chosen as a control to make sure that we would obtain the same results with a different task and no reference to the causal origin of the shape’s features. Before the experiment, observers were given eight practice trials with different base shapes in order to familiarize them with the task. The complete experiment consisted of 120 trials (10 shapes times 12 repetitions).

### Experiment 2

#### Observers

A new group of 14 observers participated in Experiment 2; other details were identical to those in Experiment 1.

#### Stimuli

**Figure 4:**
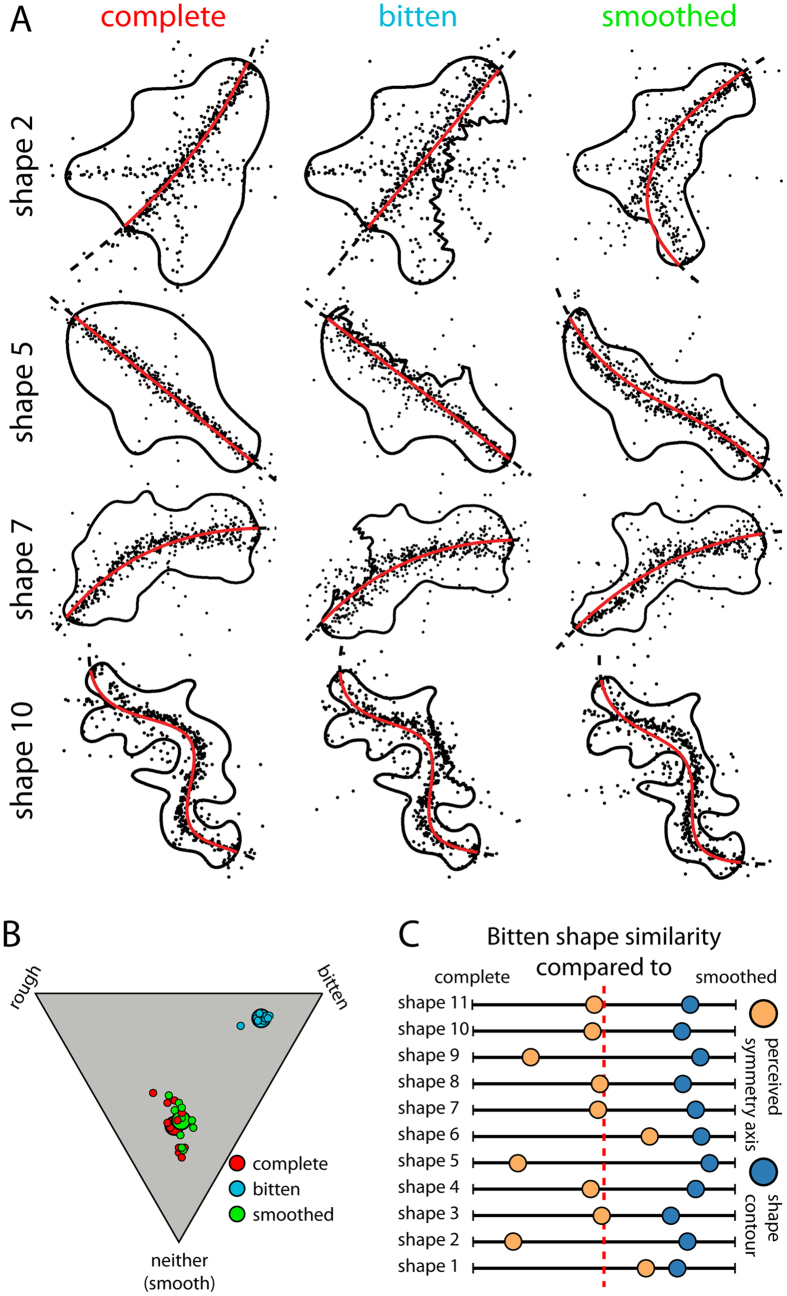
Results of Experiment 2. (**A**) Four shape examples shown in all three conditions (complete, bitten and smoothed). Black dots (N = 560 per shape and condition) represent individual responses and red lines non-linear PCA fits to the individual distributions. (**B**) Ratings whether shapes are bitten, rough or neither of the two (smooth). Small dots are individual observers’ responses collapsed across four repetitions and large dots represent the overall mean across all observers and repetitions. (**C**) Similarity of responses (orange) and shape contour (blue) of bitten shapes to their complete and smoothed counterparts. The more similar the features between conditions, the nearer a point towards the corresponding side of the line.

Stimuli were 33 different irregular 2D shapes, produced from 11 base shapes manually drawn in illustrator (see contours in Figs 4A and S2). Each base shape existed in a non-transformed version (complete), in a version where a part of the shape was removed by another shape (bitten) and a version were all tangent discontinuities in the outline of the bitten version were smoothed (smoothed). The lengths of the longest axes of base shapes 1–11 were varying between 9.95° and 11.94° of visual angle. All stimuli are available for download at http://dx.doi.org/10.5281/zenodo.159923.

#### Procedure

The experimental setup was the same as in Experiment 1. Each trial consisted of two parts—(i) a judgment about the perceived transformation and (ii) the indication of shape symmetry as described for Experiment 1. In the first part we confirmed that observers interpreted the different versions of the objects as expected using a rating task. On each trial participants viewed a single shape at a random orientation on one half of the screen and reported the extent to which it appeared rough, bitten or smooth. They did this by moving a dot within a triangular region, whose vertices were labeled ‘rough’, ‘bitten’ and ‘smooth (neither of the two)’. The position of the dot within the triangle indicated the relative strengths of the different interpretations, in a way that forced the different interpretations to trade off against one another (i.e., it was assumed that an object could not appear both strongly smooth and strongly rough at the same time). Left click confirmed their response, removed the triangular scale and centered the shape on the screen for the second task, in which observers then indicated the symmetry of the same shape by placing ten dots along the perceived symmetry axis (see Experiment 1). Experiment 2 consisted of total of 132 trials (11 shapes times three transformations times four repetitions).

### Experiment 3

#### Observers

Two new groups of 12 observers participated in Experiment 3; other details were identical to Experiment 1. One group completed the front-back task (see below), the other group completed a rating task identical to that of Experiment 2.

#### Stimuli

**Figure 5:**
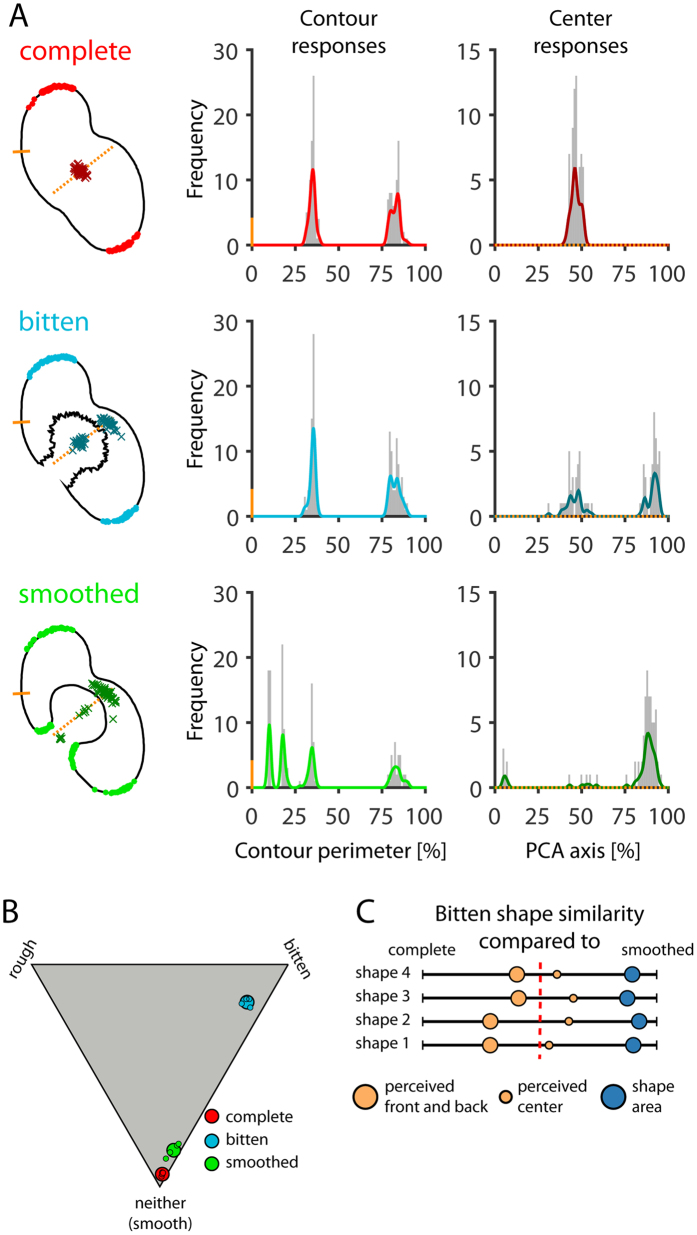
Results of Experiment 3. (**A**) Example responses to complete (red), bitten (blue) and smoothed (green) versions of shape 1. First column: Colored dots (N = 144 per shape and condition) are individual responses in the front-back task, colored crosses (N = 72 per shape and condition) are responses in the center task. Second column: Response distributions in the front-back task, sampled from the position of the short solid orange line counterclockwise around the contour of the complete version (bin width was 1% of contour perimeter). Third column: Response distributions in the center task, sampled along the axis of the first principal component (given by a PCA of data points pooled across all three conditions; dotted orange line) after projecting all responses unto that axis. Axis length was chosen as 110% of most distant responses on the axis (bin width was 1% of axis length). (**B**) Ratings of whether each shape was ‘bitten’, ‘rough’ or ‘neither of the two (smooth)’. Small dots are mean responses for the four shapes collapsed across six repetitions, and large dots represent the overall mean across all observers, shapes and repetitions. (**C**) Similarity of bitten shapes to their complete and smoothed counterparts, in terms of physical shape similarity (blue) and responses (orange). Relative similarity is indicated by proximity to either the ‘complete’ end or the ‘smoothed’ end of the scale.

Stimuli were four new irregularly shaped asymmetric objects, each of which was created by the same method as in Experiment 2 to be either (i) complete, (ii) bitten, or (iii) smoothed (see contours in Fig. 5 and Figs S3–5). The lengths of the longest axes of base shapes 1–4 were 9.40°, 9.85°, 9.05°, and 8.59° of visual angle. All stimuli are available for download at http://dx.doi.org/10.5281/zenodo.159923.

#### Procedure

The experimental setup and the rating task were the same as in Experiment 2. Before the start of the experiment, participants were provided with printouts of four familiar shapes (silhouettes of a bottle, a winding snake, a key, and a fish turning a corner) with marked fronts and backs, followed by a training task in which they were asked to indicate the front and back on printouts of six other familiar shapes (silhouettes of a jumping dolphin, a leaf, a seagull, a wheel wrench, and a partially-opened cutthroat razor). This training was intended to clarify the concept of front and back of 2D shapes, including those with curved central axes. No feedback was provided.

In the following front-back task, observers completed two blocks. First, they indicated the front and back of each shape by placing two dots on its contour. Second, they indicated the center of each shape by placing a single dot. Other details were the same as in Experiments 1 and 2. Importantly, we did not provide any explicit verbal instructions other than asking observers to identify the front, back, and center of the given shape by placing dots. Each block consisted of 72 trials (four shapes times three transformations times six repetitions)^67^,^68^,^69^.

## Additional Information

**How to cite this article**: Spröte, P. *et al*. Visual perception of shape altered by inferred causal history. *Sci. Rep.* **6**, 36245; doi: 10.1038/srep36245 (2016).

**Publisher’s note:** Springer Nature remains neutral with regard to jurisdictional claims in published maps and institutional affiliations.

## Supplementary Material

Supplementary Information

## Figures and Tables

**Figure 1:**
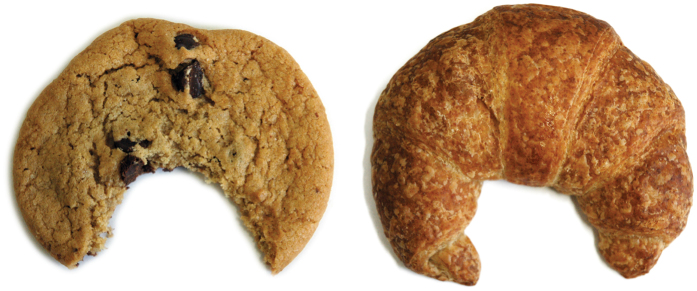
Example of different causal origins. Even though both the cookie and the croissant possess similar concavities, we readily assign different causal origins to them. The cookie appears bitten while the croissant appears bent. Reprinted from refs 53 and 70, with permission from Elsevier.
