# Supplementary material for: Visual perception of shape altered by inferred causal history
Source: Sci Rep. 2016 Nov 8;6:36245. doi: 10.1038/srep36245 (PMC5099969; doi:10.1038/srep36245)
Supplement: Supplementary Information [file srep36245-s1.pdf]

Visual perception of shape altered by inferred causal history

Patrick Spröte[1,+], Filipp Schmidt[1,+,*], and Roland W. Fleming[1]

[1]Justus-Liebig-University Giessen

[+] equal contribution

* corresponding author

Supplementary Figures

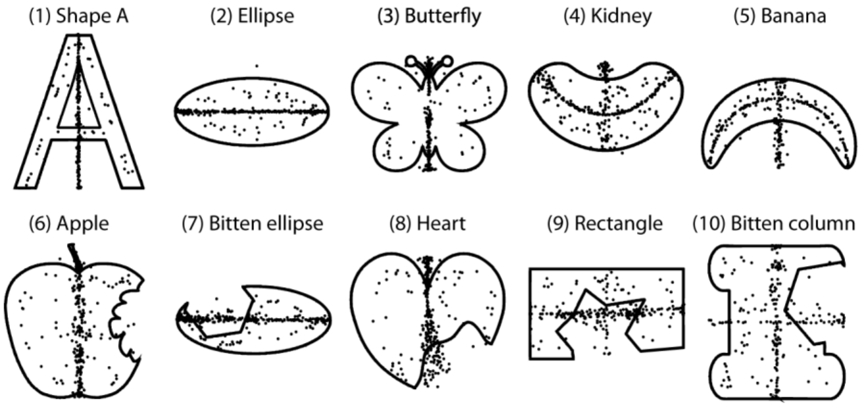

Figure S1. Results of the control experiment for Experiment 1. Black dots (N = 300 per shape) represent individual responses from n = 6 observers. On each trial participants were presented with a 'fixed' dot at some point in the shape, and had to move an 'adjustable' dot 'to the symmetrical location on the other side of the shape'. Midway between the two dots was a small line element, oriented perpendicular to the imaginary line joining the two, which participants were instructed should lie along the apparent symmetry axis of the object, if the adjustable dot were placed appropriately. Black dots in the figure represent the location of this line element for each of the dot pairs presented to participants. Unlike, in the main experiment no instructions were given regarding how to respond when the features were interpreted in different ways.

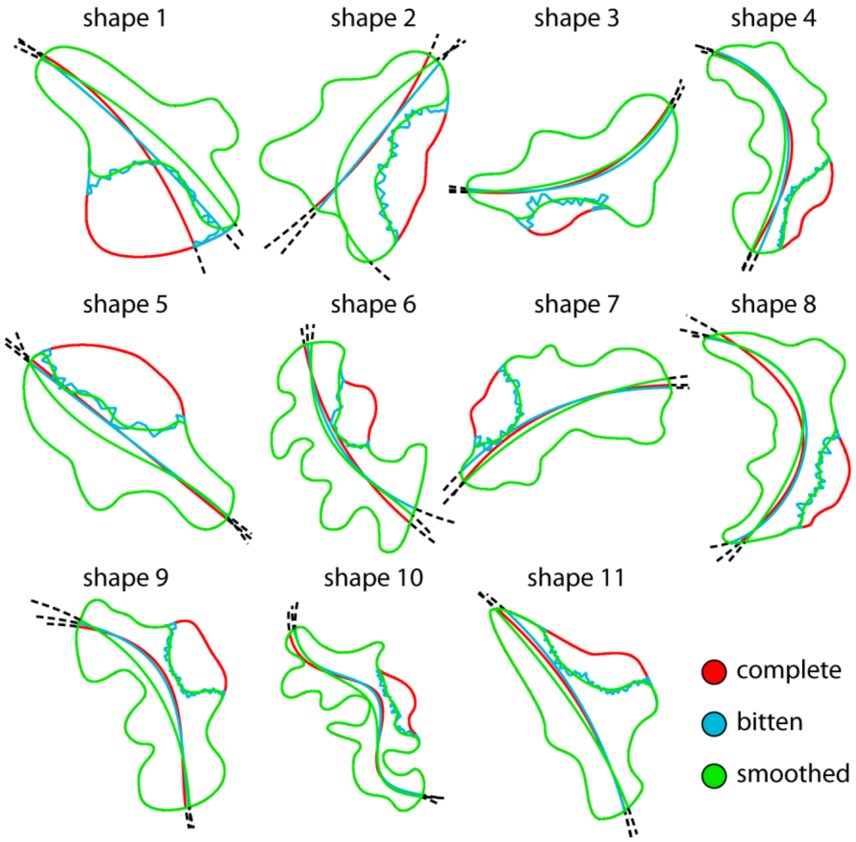

Figure S2. All three transformations of individual shapes from Experiment 2 superimposed. Colors red, blue and green correspond to complete, bitten and smoothed version of a shape respectively. Solid curves represent principal axes derived from observers' response using non-linear PCA. Responses to bitten shapes were more similar to complete shapes than to the smoothed versions for all shapes except 1 and 6 (see also Figure 4).

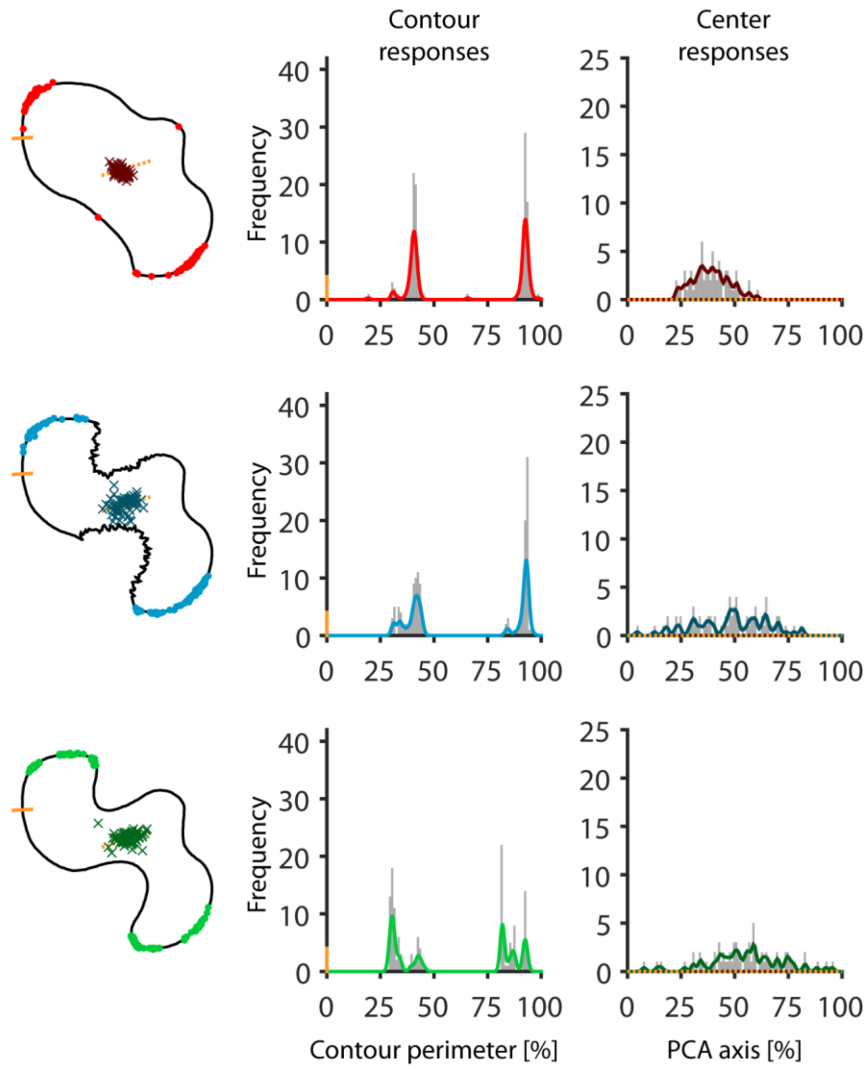

Figure S3. Results for shape 2 in Experiment 3. For descriptions see Figure 5A.

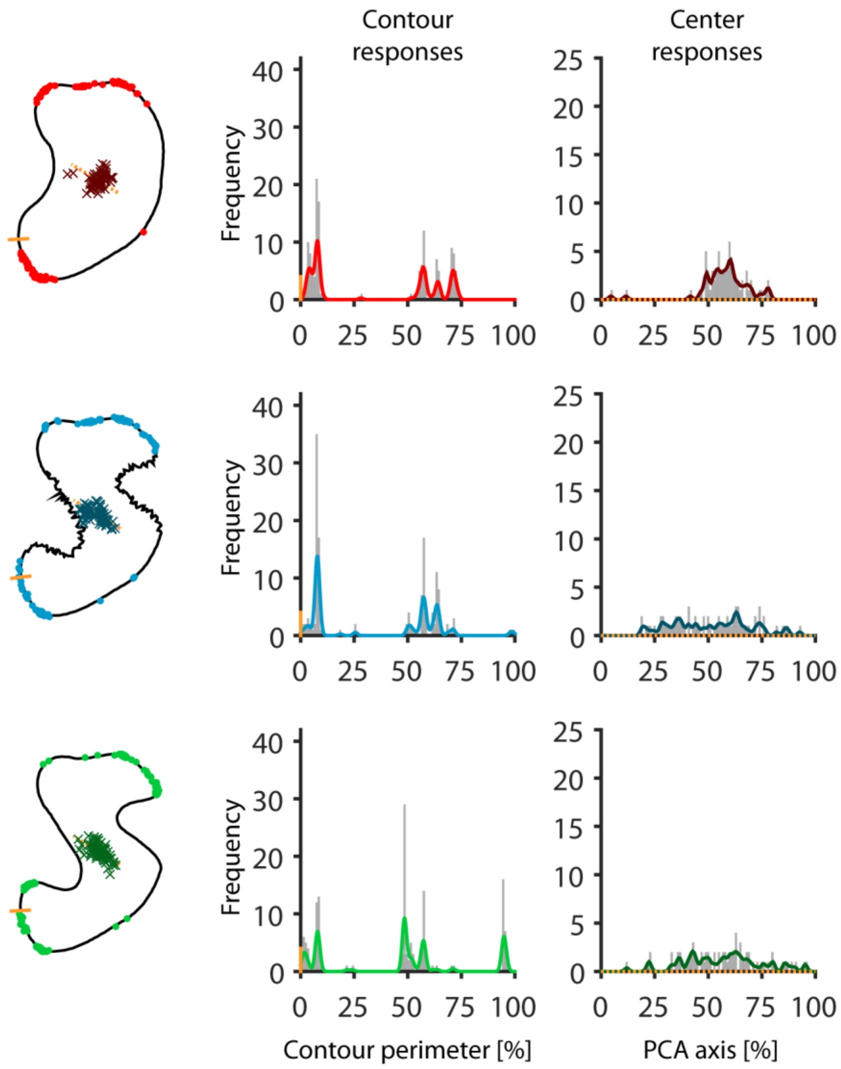

Figure S4. Results for shape 3 in Experiment 3. For descriptions see Figure 5A.

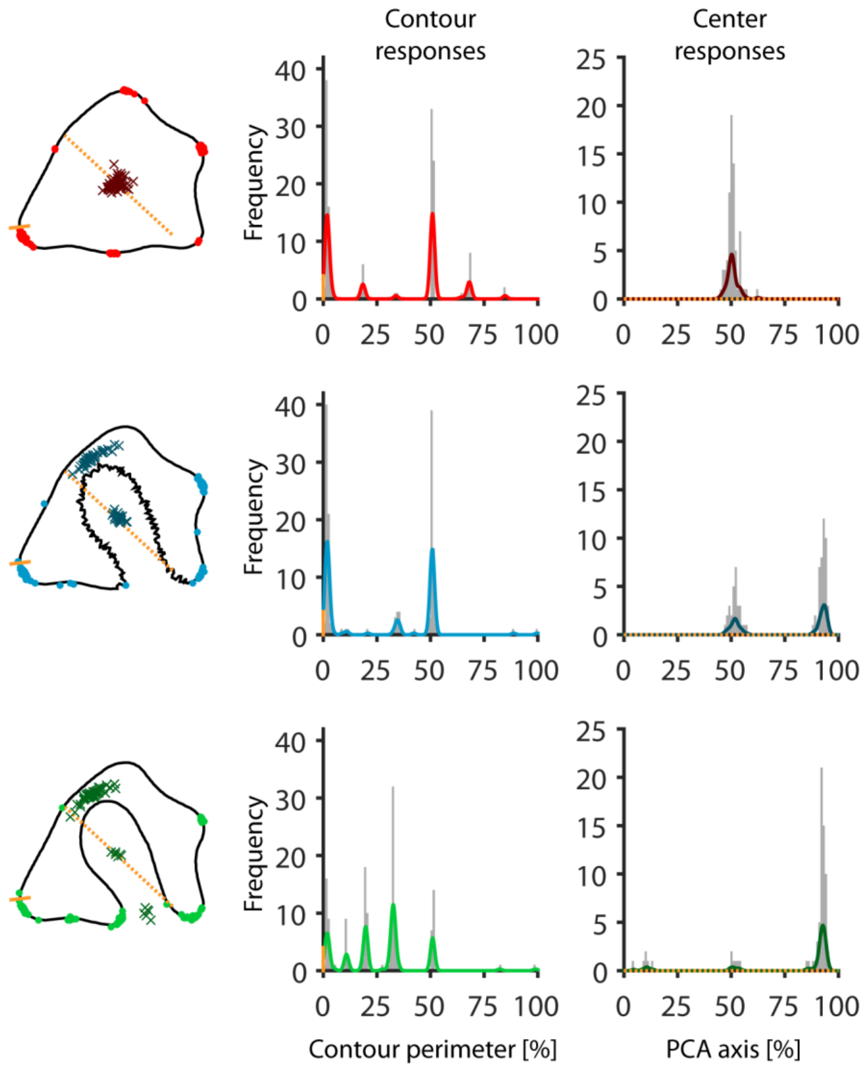

Figure S5. Results for shape 4 in Experiment 3. For descriptions see Figure 5A.
